# Exploitation of the Genetic Variability of Diverse Metric Traits of Durum Wheat (*Triticum turgidum* L. ssp. *durum* Desf.) Cultivars for Local Adaptation to Semi-Arid Regions of Algeria

**DOI:** 10.3390/plants13070934

**Published:** 2024-03-23

**Authors:** Zine El Abidine Fellahi, Tahar Boubellouta, Abderrahmane Hannachi, Haroun Belguet, Nasreddine Louahdi, Amar Benmahammed, Aleksandra O. Utkina, Nazih Y. Rebouh

**Affiliations:** 1 Department of Agronomic Sciences, Faculty of Natural, Life and Earth Sciences and the Universe, University of Mohamed El Bachir El Ibrahimi, Bordj Bou Arreridj 34030, Algeria; 2 Valorization of Natural Biological Resources Laboratory (VNBR), University of Ferhat Abbas Setif-1, Setif 19000, Algeria; benmahammeda@yahoo.com; 3 Department of Biological Sciences, Faculty of Natural, Life, Earth and Universe Sciences, University Mohamed El Bachir El Ibrahimi, Bordj Bou Arreridj 34030, Algeria; t.boubellouta@univ-bba.dz; 4 Characterization and Valorization of Natural Resources Laboratory (CVNRN), University Mohamed El Bachir El Ibrahimi, Bordj Bou Arreridj 34030, Algeria; 5 National Agronomic Research Institute of Algeria (INRAA), Setif Research Unit, Setif 19000, Algeria; 6 Experimental Farm, Field Crop Institute (ITGC), Farm Road-BP03, Setif 19000, Algeria; harounbelguet@gmail.com (H.B.); n.louahdi@gmail.com (N.L.); 7 Department of Ecology and Plant Biology, Faculty of Natural and Life Science, University of Ferhat Abbas Setif-1, Setif 19000, Algeria; 8 Department of Environmental Management, Institute of Environmental Engineering, RUDN University, 6 Miklukho-Maklaya St., 117198 Moscow, Russia; utkina-ao@rudn.ru

**Keywords:** *Triticum durum*, genotypic correlation, path analysis, Mahalanobis distance, adaptation, performance, semi-arid

## Abstract

Abiotic stresses pose significant challenges to wheat farming, yet exploiting the genetic variability within germplasm collections offers an opportunity to effectively address these challenges. In this study, we investigated the genetic diversity of key agronomic traits among twenty durum wheat cultivars, with the intention to pinpoint those better suited to semi-arid conditions. Field trials were conducted at the ITGC-FDPS Institute, Setif, Algeria, during the winter season of 2021/22. A completely randomized design was used with three replicates. Statistical analyses revealed significant variation among the genotypes for most of the studied traits, with some cultivars exhibiting a superior performance in a stressful environment. Notably, traits like the number of grains per spike (NGS) and the grain yield (GY) displayed high genotypic coefficients of variation (CVg). Except for membrane thermostability (MT) and biological yield (BY), the majority of the assessed traits exhibited moderate-to-high heritability estimates. Genotypic and phenotypic correlation studies have confirmed the importance of many yield-related traits in the expression of GY. The harvest index (HI) underscored the highest genotypic direct effect on GY, followed closely by spike number (SN), serving as consistent pathways through which most of the measured traits indirectly influenced GY. The cluster analysis categorized the durum wheat cultivars into seven distinct clusters. The largest inter-cluster distance was observed between clusters G3 and G4 (D^2^ = 6145.86), reflecting maximum dissimilarity between the individuals of these clusters. Hybridizing divergent clusters may benefit future breeding programs aiming to develop potential durum wheat varieties through cross combinations. This study’s findings contribute to sustainable agriculture efforts by facilitating the selection of genotypes with enhanced resilience and productivity, particularly for cultivation in challenging semi-arid regions.

## 1. Introduction

Durum wheat (*Triticum turgidum* L. ssp. *durum* Desf.) is an important cereal crop, particularly in regions with limited water availability [1,2]. Algeria, being a country with diverse climatic conditions, including harsh environments, faces challenges in durum wheat cultivation due to water scarcity and other biotic and abiotic stresses [3]. To address these challenges and enhance durum wheat production, researchers and breeders focus on exploiting the genetic variability of diverse quantitative and some qualitative traits within old and modern germplasm [4,5,6,7]. 

Durum wheat germplasm represents a diverse collection of genetic resources, which includes different varieties, landraces, and wild relatives [8,9]. This genetic diversity possesses a range of traits, including highly heritable characteristics like heading date, plant height, and kernel weight, but also a large number of quantitative traits, such as yield potential, drought tolerance, disease resistance, and nutrient use efficiency, which exhibit continuous variation [10,11,12]. 

Through the systematic evaluation of diverse durum wheat germplasm collections, breeders have identified cultivars that possess desirable traits. This can be based on phenotypic traits like plant height, flowering time, grain yield, and quality characteristics, as well as genotypic traits revealed through molecular markers [13,14,15]. After crossing cultivars carrying desirable traits, breeders—through traditional breeding methods—select superior progenies over multiple generations [16]. Molecular breeding approaches, such as marker-assisted selection and genomic selection, facilitate the identification and utilization of specific genes or genomic regions associated with target traits [17,18]. 

Genetic and non-genetic parameters, such as phenotypic (*CV_p_*) and genotypic coefficient of variation (*CV_g_*), along with heritability (*h^2^_bs_*), serve as valuable biometric indicators for assessing the genetic variation and adaptability within a germplasm collection. They provide insights into the relative contributions of genetic and environmental factors to observed variations in specific traits among individuals [19,20,21].

Because grain yield is a polygenic complex trait that is controlled by many factors, and is extremely influenced by environmental variation, selection for high-yielding genotypes should not be based on yield only, but other yield-contributing traits should be taken into consideration during the selection process [22,23]. Hence, knowledge of the degree of relationship between yield and different yield-related components can identify the traits that could be used as indirect selection criteria, increasing the efficiency of the selection process. The genotypic and phenotypic correlations are concepts used by breeders to describe the association between the genetic makeup (genotype) and the observable traits (phenotype) of individuals within a population [24]. Genotypic correlation focuses on the genetic basis of traits, while phenotypic correlation considers the observable expression of these traits in individuals, influenced by both genetic and environmental factors [25]. 

In fact, the correlation coefficient only quantifies the link between each pair of traits and does not truly indicate the relative importance of secondary traits on the yield. Path coefficient is a common approach used in the field of crop breeding, where researchers are interested in understanding the complex relationships between grain yield and its related traits in order to identify those with significant effects on yield materialization to be used as selection criteria [26]. This method has the advantage of portioning the correlation coefficient into direct and indirect relationships among a set of variables, helping to understand the complex interplay between the different factors in a theoretical model [26]. The direct effect refers to the influence of one trait on another without the mediation of any other traits, whereas the indirect effect considers the impact of one trait on another that is mediated through one or more intervening traits in the model [27]. According to Boulelouah et al. (2022) [28], both correlation and path coefficient techniques could achieve knowledge regarding the proper cause-and-effect relationship between yield and yield components.

By understanding the morpho-genetic dissimilarity among diverse cultivars within the available germplasm, breeders can strategically choose parents for crossing with the goal of maximizing the potential for genetic gain. Cluster analysis is a statistical method used in the context of genetic diversity studies to classify cultivars of germplasm collections in specific heterotic groups [29]. In this context, the generalized Mahalanobis (1936) distance method provides greater precision when selecting genotypes for future crosses. It was largely used as a consistent measure of dissimilarity in various crop species, including durum wheat [30,31]. This information is valuable for both understanding the genetic structure of the population and for guiding breeding strategies to harness the genetic diversity for crop improvement.

In view of this, the present investigation was conducted to explore the genetic divergence and variability present within a durum wheat germplasm collection through the phenotypic evaluation of various desirable characteristics of particular importance for local adaptation to semi-arid regions of Algeria. This evaluation allows breeders to identify individuals as potential parents for future breeding programs targeting the development of wheat varieties with enhanced productivity and resilience.

## 2. Results 

### 2.1. Sources of Variation Analysis

The results showed highly significant differences (*p* ≤ 0.01) among the tested genotypes for the majority of traits subjected to one-way analysis of variance (ANOVA), except for MT and BY, which were non-significant (Table 1). 

### 2.2. Mean Performance of Genotypes

The mean performance of 20 durum wheat genotypes for 15 morpho-physiological and agronomic traits at the Setif location is presented in Table 2. The HD elapsed between 128.0 days in Ofanto and 138.0 days in Guemgoum Rkhem, with a mean of 132.4 days. The mean CC was 43.6 CCI. The minimum CC was 29.3 CCI, which was exhibited by Bousselam, whereas the maximum value of CC was recorded in Simeto, at 54.3 CCI. The CT varied between 29.7 °C in Megress and 36.9 °C in Bousselam, with an average value of 32.8 °C. The mean RWC was 70.6%. Belikh02 was the cultivar with the lowest RWC, i.e., 62.3%, whereas Mohamed Ben Bachir was the genotype with the highest water content in the leaves, at 81.7%. Despite the wide range observed for MT, the difference among the genotypes for this trait was not significant. The mean value of MT assessed in the wheat genotypes was 80.6%. Ofanto had the lowest cell MT, which was 51.4%, whereas Amar06 had the highest MT, which was 95.2%. 

The mean FLA was 30.3 cm^2^. Belikh02 had the smallest leaves, at 21.9 cm^2^, whereas Waha had the largest leaves, at 36.3 cm^2^. The mean PH was 79.6 cm. Mexicali75 was the shortest cultivar, with a plant height of 69.3 cm, whereas Guemgoum Rkhem was the tallest genotype, with a PH of 108.7 cm. The mean value of all of the genotypes for SN was 378.7 spikes m^−2^. The lowest mean value of SN (225.0 spikes m^−2^) was obtained in the Guemgoum Rkhem cultivar, while the highest mean value of this trait (496.7 spikes m^−2^) was exhibited by Waha. The overall average SW was 4.1 t ha^−1^, with a range from 2.3 (Guemgoum Rkhem) to 5.2 t ha^−1^ (Wahbi). The mean value of TKW was 30.1 g. The lowest kernel weight (21.1 g) was exhibited by Bousselam, while the highest TKW (41.0 g) was recorded for Boutaleb. The minimum NGS recorded was 4.3 grain spike^−1^ for Vitron, whereas a maximum of 15.8 grains spike^−1^ was found for Manssourah, with an average value of 8.8 grains spike^−1^. The mean GY was 1.9 t ha^−1^. Zb/Fl had the lowest GY, at 1.1 t ha^−1^, whereas Manssourah had the highest GY, at 3.2 t ha^−1^. The minimum BY recorded was 7.2 t ha^−1^ for Belikh02, whereas a maximum of 12.3 t ha^−1^ was registered for Saoura, with a mean value of 10.9 t ha^−1^. Mohamed Ben Bachir had the lowest HI, i.e., 9.6% t ha^−1^, whereas Manssourah displayed the highest HI, i.e., 32.1%, with an average HI value of 17.9%.

### 2.3. Estimation of Components of Variation

The estimates of genetic and non-genetic parameters for the measured traits in the wheat cultivars are illustrated in Table 3. The phenotypic coefficient of variation (*CV_p_*) values varied between 1.99 for HD and 38.95% for GY. The genotypic coefficient of variation (*CV_g_*) ranged from 1.99 for HD to 32.62% for HI. The *CV_p_*, as well as the *CV_g_*, values were categorized as low (<10%), moderate (10 to 20%), and high (>20%) [32]. A high *CV_p_* indicates a considerable variation for the trait within the population, which may be due to genetic differences, environmental influences, or their interactions. In this study, next to GY (37.63%), HI (37.63%), NGS (32.29%), SN (26.64%), SW (26.34%), CC (23.14%), and TKW (20.22%) also presented high *CV_p_* estimates. By contrast, low values of *CV_p_* were found in HD (1.99%), CT (5.76%), and RWC (6.31%), whereas the remaining recorded traits exhibited moderate values of *CV_p_*.

### 2.4. Relationships among Measured Traits

**Figure 1:**
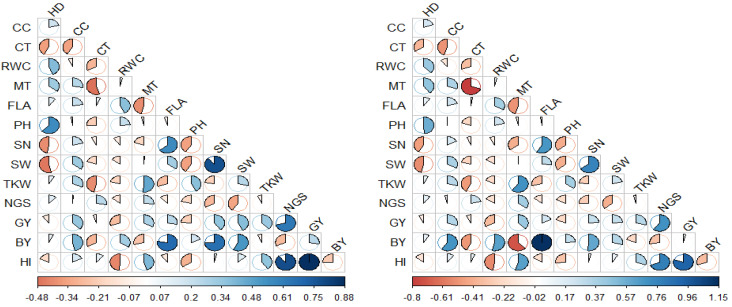
Correlogram representing phenotypic (**left**) and genotypic (**right**) correlation coefficients among the 13 measured traits in the durum wheat cultivars. HD: Heading date, CC: Chlorophyll content, CT: Canopy temperature, RWC: Relative water content, MT: Membrane thermostability, FLA: Flag leaf area, PH: Plant height, SN: Spike number, SW: Spike weight, TKW: Thousand kernel weight, NGS: Number of grains per spike, GY: Grain yield, BY: Biological yield, HI: Harvest index.

Figure 1 represents the phenotypic (*r_p_*) and genotypic (*r_g_*) correlation coefficients among the 13 morpho-physiological and agronomic traits measured in the durum wheat cultivars. At both genotypic and phenotypic levels, GY showed significant to highly significant and positive relationships with CC (*r_p_* = 0.332 ** and *r_g_* = 0.343 **), SN (*r_p_* = 0.443 ** and *r_g_* = 0.270 *), SW (*r_p_* = 0.497 ** and *r_g_* = 0.270 *), TKW (*r_p_* = 0.337 ** and *r_g_* = 0.332 **), NGS (*r_p_* = 0.558 ** and *r_g_* = 0.693 **), and HI (*r_p_* = 0.833 ** and *r_g_* = 0.926**). BY, in addition to its relation to GY, it had other strong correlations, including positives with CC (*r_p_* = 0.256 * and *r_g_* = 0.628 **), FLA (*r_p_* = 0.429 ** and *r_g_* = 1.152 **), SN (*r_p_* = 0.739 ** and *r_g_* = 0.599 **), and SW (*r_p_* = 0.651 ** and *r_g_* = 0.332 **); and a negative correlation with NGS (*r_p_* = −0.262 * and *r_g_* = −0.326 *). As discussed above, the flag leaf area and the biological yield were largely influenced by similar environmental conditions (Table 3); therefore, the environmental factors that affected both traits may explain the overestimation of the genetic correlation coefficient value. On the other hand, HI demonstrated significant negative correlations with RWC (*r_p_* = −0.270 * and *r_g_* = −0.552 **) and PH (*r_p_* = −0.287 * and *r_g_* = −0.311 *), and positive associations with TKW (*r_p_* = 0.336 ** and *r_g_* = 0.357 **) and NGS (*r_p_* = 0.762 ** and *r_g_* = 0.792 **).

Worthy of note is the negative correlation of SN with HD (*r_p_* = −0.342 ** and *r_g_* = −0.474 **), PH (*r_p_* = −0.290 * and *r_g_* = −0.416 **), and NGS (*r_p_* = −0.292 * and *r_g_* = −0.288 *); and the positive correlations between SN and FLA (*r_p_* = 0.458 ** and *r_g_* = 0.671 **) and SN and SW (*r_p_* = 0.790 ** and *r_g_* = 0.762 **). The SW also showed negative relationships with HD (*r_p_* = −0.384 ** and *r_g_* = −0.531 **) and PH (*r_p_* = −0.293 * and *r_g_* = −0.414 **), and a positive correlation with FLA (*r_p_* = 0.307 * and *r_g_* = 0.299 *). The HD, in addition to its negative relations with SN and SW, showed highly significant correlations, including a negative correlation with CT (*r_p_* = −0.336 ** and *r_g_* = −0.381 **) and positive correlations with RWC (*r_p_* = 0.321 * and *r_g_* = 0.438 **) and PH (*r_p_* = 0.530 ** and *r_g_* = 0.570 **). The TKW exhibited a significant negative relationship with CT (*r_p_* = −0.490 ** and *r_g_* = −0.286 *) and significant positive correlations with MT (*r_p_* = 0.286 * and *r_g_* = 0.699 **) and PH (*r_p_* = 0.326 * and *r_g_* = 0.390 **). Furthermore, MT significantly correlated with CT (*r_p_* = −0.259 * and *r_g_* = −0.804 **) and FLA (*r_p_* = −0.361 ** and *r_g_* = −0.512 **). FLA and RWC were positively inter-correlated (*r_p_* = 0.358 ** and *r_g_* = 0.382 **) at both genotypic and phenotypic levels.

At the phenotypic level, GY presented significant to highly significant positive relationships with FLA (*r_p_* = 0.272 * and *r_g_* = 0.210 ^ns^) and BY (*r_p_* = 0.390 ** and *r_g_* = 0.045 ^ns^). Similarly, the data regarding the TKW measurements showed a significant positive correlation with SW (*r_p_* = 0.278 * and *r_g_* = 0.238 ^ns^). At the genotypic level, the GY showed strong correlations, including a negative correlation with RWC (*r_p_* = −0.152 ^ns^ and *r_g_* = −0.366 **) and a positive correlation with MT (*r_p_* = 0.651 ** and *r_g_* = 0.332 ^ns^). The BY significantly correlated with most of the physiological traits, including CT (*r_p_* = −0.176 ^ns^ and *r_g_* = −0.497 **), RWC (*r_p_* = 0.111 ^ns^ and *r_g_* = 0.625 **), and MT (*r_p_* = −0.059 ^ns^ and *r_g_* = −0.721 **). The BY also displayed a positive genotypic correlation with PH (*r_p_* = 0.085 ^ns^ and *r_g_* = 0.281 *) and a strong negative correlation with HI (*r_p_* = −0.152 ^ns^ and *r_g_* = −0.354 **). The latter also demonstrated a highly significant positive association with MT (*r_p_* = 0.241 ^ns^ and *r_g_* = 0.629 **). Other significant correlations were also observed among the measured traits. For example, negative correlations of SN with MT (*r_p_* = −0.033 ^ns^ and *r_g_* = −0.409 **) and TKW (*r_p_* = −0.113 ^ns^ and *r_g_* = −0.269 *) were found. The latter trait also demonstrated other important correlations, including a positive correlation with CC (*r_p_* = 0.222 ^ns^ and *r_g_* = 0.322 *) and a negative with correlation FLA (*r_p_* = −0.200 ^ns^ and *r_g_* = −0.326 *). The CC exhibited strong positive relationships with MT (*r_p_* = 0.186 ^ns^ and *r_g_* = 0.353**) and SW (*r_p_* = 0.241 ^ns^ and *r_g_* = 0.378 **) and a negative correlation with CT (*r_p_* = −0.186 ^ns^ and *r_g_* = −0.508 **). The CT and RWC were negatively inter-related (*r_p_* = −0.221 ^ns^ and *r_g_* = −0.349 **). The data regarding the MT measurements showed highly significant positive correlations with HD (*r_p_* = 0.218 ^ns^ and *r_g_* = 0.460 **) and NGS (*r_p_* = 0.105 ^ns^ and *r_g_* = 0.398 **). 

### 2.5. Estimate of Direct and Indirect Effects at a Genotypic Level

**Figure 2:**
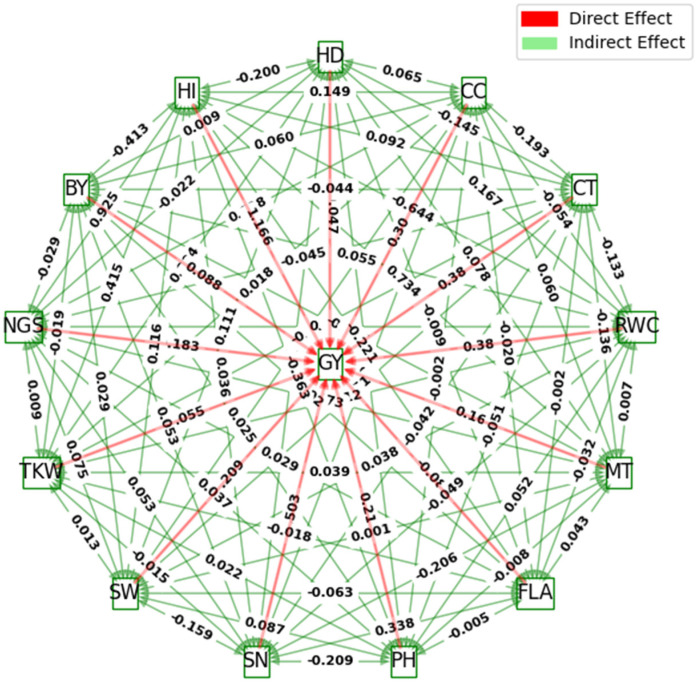
Genotypic path diagram representing the direct and indirect effects of 14 measured traits on grain yield in the durum wheat genotypes. HD: Heading date, CC: Chlorophyll content, CT: Canopy temperature, RWC: Relative water content, MT: Membrane thermostability, FLA: Flag leaf area, PH: Plant height, SN: Spike number, SW: Spike weight, TKW: Thousand kernel weight, NGS: Number of grains per spike, GY: Grain yield, BY: Biological yield, HI: Harvest index.

The results of the path coefficient analysis of 12 measured traits to GY are given in Table 4 and Figure 2. Dewey and Lu (1959) [26] classified path coefficient values as very high (>1.0), high (0.30–0.99), moderate (0.2–0.29), low (0.1–0.19), and negligible (0.00–0.09). Based on this, the harvest index (1.166) and RWC (0.381) exhibited very high and high positive direct effects on GY, respectively. Traits such as SN (0.503), CT (0.380), and CC (0.302) exerted moderate positive direct effects on GY, similar to their genotypic correlations with this primary trait (Table 4; Figure 1). The PH (0.215) and MT (0.169) expressed moderate-to-low direct effects on GY. Conversely, SW (−0.209), next to NGS (−0.183), yielded the first and second highest negative direct effects on GY, respectivley. While HD, FLA, and BY had non-significant associations with GY at a genotypic level, it has been stated in Table 4 and Figure 2 that the SW and NGS demonstrated positive and statistically significant correlations with the grain yield per hectare.

The NGS (0.925), MT (0.734), and TKW (0.415) depicted the highest positive indirect effects on GY via HI. Furthermore, SW (0.383), FLA (0.338), and BY (0.301) exhibited high positive indirect effects on GY through SN. The BY expressed moderate positive indirect effects on GY through RWC (0.238) and CC (0.206). On the other hand, the RWC (−0.644), followed by the BY (−0.413), PH (−0.363), FLA (−0.221), and HD (−0.200), demonstrated high-to-moderate negative indirect effects on GY via HI. Similarly, the HD (−0.239), next to the PH (−0.209) and MT (−0.206), had moderate negative indirect effects on GY through SN. MT, via CT (−0.306), and HI, via RWC (−0.210), also exerted high and moderate negative indirect effects on GY, respectively. The rest of the indirect effects on GY were low-to-negligible, as shown in Table 4 and Figure 2.

### 2.6. Divergence among Wheat Cultivars

The 20 durum wheat cultivars were classified into seven distinct clusters or groups, namely, G1, G2, G3, G4, G5, G6, and G7 (Table 5). Eight cultivars were found in the first cluster (G1), accounting for 40% of total genotypes, followed by four cultivars classified in the second cluster (G2), with a relative contribution of 20% to the genetic diversity of the durum wheat germplasm. The third cluster (G3) contained three genotypes, while the fourth cluster (G4) was formed by two cultivars only. Besides these four clusters, the remaining three clusters only had one cultivar (Table 5). 

**Figure 3:**
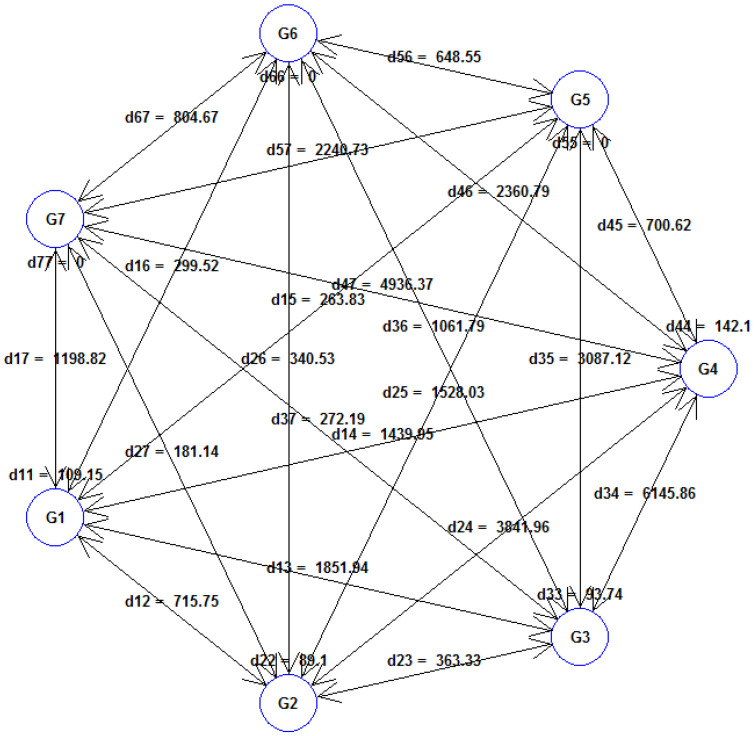
Clustering formed by Tocher’s method representing the average intra- and inter-cluster distances for the studied traits in the durum wheat cultivars.

Figure 3 illustrates the average distances within and between the seven clusters formed by Tocher’s method based on Mahalanobis Euclidean^2^ distance. The average inter-cluster distance was observed at maximum (*D^2^* = 6145.86) between cluster G3 and cluster G4. The lowest inter-cluster distance (*D^2^* = 181.14) was recorded between clusters G2 and G7, which exhibited more genetic similarity. The maximum intra-cluster distance was obtained in cluster G4 (*D^2^* = 142.10), followed by cluster G1 (*D^2^* = 109.16), indicating that the cultivars belonging to these clusters were far diverged from those within clusters G2 (*D^2^* = 89.10) and G3 (*D^2^* = 93.75). Clusters G5, G6, and G7 did not show any intra-cluster distance. Among the fourteen traits studied, the greatest contribution to cluster divergence was made by HD (92.33%), followed by plant height (2.08%), thousand kernel weight (1.61%), and spike weight (1.52%).

The average of the studied traits for each cluster has been represented in Table 6. Accordingly, cluster G4 was characterized by having the highest cluster mean for days to 50% heading (137.5 days), while cluster G3 had the lowest HD mean (128.7 days). G3 and G4 exhibited, respectively, the lowest (67.0) and the highest (75.4 CCI) chlorophyll content in the leaves. The cultivars grouped in cluster G1 showed the highest CT (47.2%), GY (2.4 t ha^−1^), BY (11.6 t ha^−1^), and HI (21.2%) values.

Cluster G2 showed the highest SN (411.3 spikes m^−2^), whereas the lowest SN (240.8 spikes m^−2^) was observed in cluster G4, which had also the longest plant stature (104.7 cm) and the lowest SW (2.4 t ha^−1^), GY (1.1 t ha^−1^), and HI (9.8%) values. Cluster G5 is characterized by the shortest plants (73.3 cm) and the highest RWC (91.2%) and NGS (10.1 grains spike^−1^). Cluster G6 had the coolest canopy cover (29.3 °C), the largest leaves (33.8 cm^2^), the highest MT (36.9%), and the smallest grain size (21.1 g). Cluster G7 had the lowest cluster mean for MT (31.5%), the smallest leaves (24.7 cm^2^), the lowest spike fertility (4.3 grains spike^−1^), and the lowest BY (9.2 t ha^−1^). This cluster is characterized also by the highest SW (5.0 t ha^−1^) and the largest grain size (40.1 g). 

## 3. Discussion

The results of the present study have revealed considerable variability in plant material, suggesting ample opportunities for wheat breeders to exploit this genetic diversity in breeding programs. Previous research in the Eastern Algerian High Plateaus supports these findings [33,34,35]. Researchers have noticed a wide variation in physiological, yield, and yield-related traits. The lack of significant differences in aboveground biomass may be due to water stress and increased temperatures at the post-anthesis growth stage, reducing cell division and elongation, leaf area, and grain filling period. The absence of significant differences in cell membrane thermostability could prompt further genetic studies by local breeders to enhance heat stress tolerance through hybridization and selection programs.

This study demonstrates that the mean trait values vary among the different genotypes of durum wheat, indicating distinct trait profiles and underlying genetic differences that influence morpho-physiological and agronomic features. This variability is crucial for selecting and breeding durum wheat varieties with desired characteristics, such as a higher yield, disease resistance, environmental stress tolerance, and enhanced nutritional content. By identifying the superior genotypes for specific traits, breeders can strategically design breeding programs to develop improved durum wheat varieties suited to various growing conditions and end-use requirements. [36]. 

This study has revealed varying levels of *CV_g_* in durum wheat germplasm, with some traits exhibiting high-to-moderate *CV_g_* values, offering opportunities for improvement through selective breeding. Conversely, the *CV_g_* estimates were low for other traits, implying limited genetic variability that may hinder traditional selective breeding methods [37]. Additionally, the difference between *CV_p_* and *CV_g_* values has highlighted the influence of environmental factors on trait variability, particularly for traits like CC, MT, FLA, and yield components, excluding TKW. In such a situation, breeders should consider this information when developing strategies to enhance genotypic stability and environmental adaptability in wheat varieties [38,39]. Apart from MT and FLA, most of the measured traits in the durum wheat germplasm displayed high genetic control (*CV_g_*/*CV_e_* > 1), making them favorable targets for breeding programs, due to their responsiveness to selection efforts. On the other hand, those traits with a variation index below one (*CV_g_*/*CV_e_* < 1) are predominantly influenced by environmental factors, rendering them less predictable across various growing conditions [40]. Consequently, indirect selection, or the consideration of environmental factors, may be necessary to effectively improve these traits [22]. The broad-sense heritability (*h^2^_bs_*) has further supported these findings, indicating traits with high genetic determinism, like HD, CT, PH, TKW, NGS, and HI, which are desirable for breeding programs, due to their amenability to selection efforts. *h^2^_bs_* was just intermediate for CC, RWC, FLA, SN, SW, and GY, suggesting that both genetic and environmental factors contribute to the observed variation in these traits. Conversely, less heritable traits pose challenges in breeding, due to their susceptibility to environmental influences. 

Bendjama and Ramdani (2022) [20] also observed high heritability for most agro-morphological traits in local durum wheat varieties grown in Algeria. However, broad-sense heritability varies depending on the population and environmental conditions [41], and it does not reveal the specific genetic mechanisms underlying the traits [19]. It only quantifies the overall genetic contribution to trait variation. Understanding the balance between genetic and environmental factors is essential for assessing the risks in genotype selection and minimizing environmental impacts on breeding outcomes.

This study has uncovered significant relationships between various traits in durum wheat, at both genotypic and phenotypic levels. The genotypic coefficients of correlation were generally larger than their corresponding phenotypic coefficients. Some significant genotypic correlations were not statistically significant at the phenotypic level, possibly due to environmental factors or sample size limitations [42]. Conversely, instances where phenotypic correlation coefficients exceeded genotypic ones could indicate gene–environment interactions, where the environmental conditions modulate the impact of the genetic factors on traits, altering the observed correlations between genotypic and phenotypic levels [43].

The results indicate that all of the cultivars had a long growth cycle with fewer fertile tillers and light spikes, but they showed tolerance to water deficit with high above-ground biomass. Despite this, they had a low harvest index and productivity. The tall cultivars had a larger grain size, due to a longer grain-filling period, allowing for more nutrient and energy accumulation, leading to higher yields under favorable conditions [44]. Conversely, the short cultivars produced heavier spikes with better fertility, more spikes per unit surface, and efficient assimilate translocation, resulting in increased biological and grain yields. This suggests a link between dwarfing genes and genes controlling spike-related traits and the plant’s capacity to allocate assimilates to grains (source–sink relationship).

Fellahi et al. (2023) [45] found that semi-dwarf wheat breeding lines carrying the Rht-D1b and Rht-B1b mutant alleles outperformed the tall lines in yield under supplemental irrigation and in spike number under rainfed conditions, along with other yield-related traits, such as harvest index and number of grains per spike, regardless of the environment. This observation is supported by the present investigation, which has revealed strong correlations between the yield and various yield attributes. Rabti et al. (2020) [5] similarly emphasized the significant influence of the spike number, spike fertility, and harvest index on the grain yield in both old and modern durum wheat varieties. These findings suggest that improving these agronomic components could substantially increase yield in the screened plant material.

According to Maeoka et al. (2020) [46], the improvement in yield of modern wheat varieties is strongly linked to reduced plant height, increased spike fertility, and higher harvest index, with no significant changes in biomass. Correlation analysis highlights the benefit of high above-ground biomass, particularly in adverse environmental conditions like drought. The cultivars with higher biological yield exhibited cooler canopy cover, better water status, and increased chlorophyll content, resulting in enhanced responses to post-anthesis water deficit and heat stress. Similar observations were made by Talebi (2011) [47], suggesting that wheat genotypes with a lower canopy temperature may have higher rates of transpiration and photosynthesis, leading to increased yield in water-stressed environments.

The genotypic path analysis results showed that the harvest index (HI) had a highly positive direct effect on grain yield (GY), consistent with findings in the existing literature [48,49]. Traits like spike number (SN), relative water content (RWC), canopy temperature (CT), and canopy cover (CC) had moderate positive direct effects on GY, reflecting their genotypic correlations with this primary trait. Despite RWC displaying a high positive direct effect on GY, it paradoxically showed a negative correlation with GY, suggesting a positive contribution to the overall crop yield. However, the negative genetic inter-correlation between RWC and GY indicates a tendency for genetic variations in RWC to be inversely related to variations in yield. This underscores the complexity of genetic interactions [50]. Plant height (PH) and maturity (MT) exhibited moderate-to-low direct effects on GY, with the effect of PH being rendered non-significant, due to the negative indirect effects of other yield components via this secondary morphological trait.

Our results suggest that more fertile tillers, along with a good plant capacity to allocate photosynthesis products (assimilates) from vegetative organs into the formed reproductive parts (grains), lead to incremented grain yield. Thus, the significant positive genotypic correlations of GY with SN and HI are predominately attributed to the direct effect of these two traits on yield per hectare. The results presented here align with the previous research findings documented in the literature [51,52,53]. TKW exerted a negligible direct effect on GY, implying the minimal impact of the grain size on the overall yield in wheat cultivars. However, the positive and significant genotypic inter-correlation between TKW and GY suggests that, while TKW may not directly influence yield, it could be associated with other factors or processes that indirectly contribute to the yield. This hidden contribution warrants further exploration for wheat improvement. 

In the scope of this study, SW, followed by NGS, had the highest negative direct effects on GY. Despite this negative impact, they both demonstrated positive and statistically significant correlations with grain yield per hectare, which implies the true relationship between these two secondary traits with the main dependent trait, i.e., GY. This observation could be attributed to the influence of common genes with pleiotropic effects [54]. While the direct effect of the trait was negative, the other effects of these shared genes could be positive for grain yield. Moreover, traits like spike weight and spike fertility might negatively affect one aspect of the plant’s physiology or morphology that directly contributes to the yield. However, compensatory mechanisms, possibly involving other traits or physiological processes, might be at play, mitigating their negative impacts. In addition, the relationship could be sensitive to environmental conditions. Under certain conditions, the negative direct effect might dominate, while under different conditions, the positive genetic correlation with yield becomes more evident. Thus, selection based on spike weight would be effective but should be practiced cautiously [38]. 

Path analysis has illustrated that NGS, MT, and TKW expressed the highest positive indirect effects on GY through HI. Additionally, SW, FLA, and BY depicted high positive indirect effects on GY via SN. BY also exhibited moderate positive indirect effects on GY via RWC and CC. These results confirm the importance of SN and HI and elucidate that the improvement of yield under drought stress during the grain filling stage could be more effective through selection. 

Tocher’s clustering partitioned the twenty durum wheat cultivars into seven distinct clusters. Notably, the maximum inter-cluster distance was found to be at maximum between clusters G3 and G4, indicating a wide dissimilarity between the cultivars in these clusters. The greatest contribution to cluster divergence was made by HD, followed by PH, TKW, and SW. This divergence may be due to historical breeding practices, geographic isolation, or other factors influencing the genetic makeup of these wheat cultivars [55]. Rabti et al. (2020) [5] have also stated that earliness had a strong influence on grain yield variability in both old and recent durum wheat germplasm.

Understanding the genetic relationships and distances between different accessions enables breeders to strategically select parents for hybridization, enhancing specific traits or overall performance [56]. Crossing genotypes from genetically divergent clusters can generate transgressive segregants, increasing the genetic variation in the offspring and facilitating the development of new potential varieties [57]. The genotypes in distant groups may possess unique or rare traits important for crop improvement, particularly in addressing challenges such as diseases, pests, or environmental stress [58]. However, the agronomic potential of those genotypes must be considered in order to develop potential varieties through cross combinations in wheat breeding programs [59,60]. The results have shown that cluster G1 could serve as a parent for developing high-yielding wheat varieties, while the presence of early heading cultivars in cluster G3 suggests the potential for developing early maturing varieties to mitigate heat waves during grain filling. The genotypes in other clusters may be selected based on their trait mean values, depending on the desired direction of selection (i.e., increase or decrease).

## 4. Materials and Methods 

### 4.1. Plant Material 

The plant material studied comprises a collection of 20 durum wheat (*Triticum turgidum* L. ssp. *durum* Desf.) cultivars (Table 7). These cultivars are selected based on their adaptability to semi-arid regions and their diverse genetic backgrounds. They represent a range of traits related to yield potential, drought tolerance, disease resistance, and quality characteristics [5,61].

### 4.2. Site Description and Experimental Design 

The field trials were conducted at the Technical Institute for Field Crops (ITGC), Demonstration and Seed Production Farm (FDPS) of Setif, during the 2021/22 crop season. The experimental farm is located at the geographical coordinates of 36°09′ N; 05°22′ E; 964 masl within the semi-arid region of Algeria, which represents the target ecological conditions. The experiment was set up under rainfed conditions in a randomized complete block design (RCBD) with three replicates. Each durum wheat variety was grown as a separate plot within the block to account for experimental variability. The plot consisted of 1.2 m wide by 5 m long plots, each containing 6 rows, with an inter-row distance of 20 cm. 

### 4.3. Soil Characteristics 

The characteristics of the soil at the experimental site are given in Table 8. Accordingly, the experimental field has a brown calcareous soil, belonging to clayey alluvial soils with an average cation exchange capacity (CEC) of 25 meq per 100 g of soil, a pH of 8.21, and 1.97% organic matter (OM) (Table 8). The percentage of soil nitrogen is 1.01‰, with 103.82 ppm of available phosphorus. The field electrical conductivity (EC) is 0.21 mmhos cm^−1^, and the exchangeable potassium is 588.7 ppm.

The sowing was carried out on 29 December, using a Hege 80 experimental plot drill at 350 seeds m^−2^ seeding rate. Adequate care for wheat growth was carried out to ensure standard agronomic practices, such as appropriate soil preparation, fertilization, and weed control. 

### 4.4. Climatic Conditions

**Figure 4:**
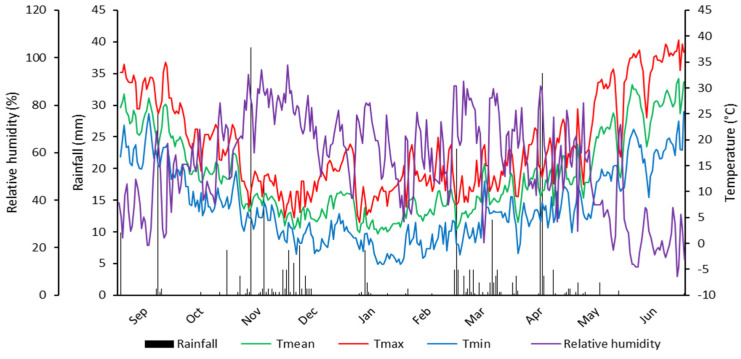
Meteorological data during the 2021/22 growth season recorded at the Setif ITGC-FDPS experimental site (https://www.tutiempo.net). accessed on 21 January 2024.

The daily rainfall amount and the maximum and minimum temperature recorded during the cropping season are displayed in Figure 4. The accumulated rainfall from 1 September to 30 June was 330.46 mm. Compared to the two previous cropping seasons, in which the total precipitation amount was, respectively, 353.06 and 320.24 mm, the 2020/21 cropping season seems to be more suitable for the yield expression of the wheat cultivars under study. However, wheat productivity is not solely determined by the amount of rainfall received, but also by its distribution during the crop cycle [62]. As shown in Figure 4, the rainfall distribution of the growing season was extremely variable. Before sowing, a good quantity of rain was observed in November, and intense precipitation with a higher total hourly amount was registered at the end of April, corresponding to the wheat anthesis growth stage. Nonetheless, the grain filling period (May to June), a sensitive stage of the wheat plant’s development, was highly affected by water stress. The rainfall amount received during the May–June period and the number of rainy days were only 6.34 mm and 7 days, respectively. The air temperature remained within the appropriate range for crop development, except for in June, in which the highest temperature was somewhat high, exceeding 35 °C. The daily mean temperatures were below 10 °C during most of the crop growing season. These climatic conditions of the field assessment cycle allowed us to screen the plant material for post-anthesis drought stress, which resulted in severe yield loss in most of the wheat cultivars tested. 

### 4.5. Data Collection

This study collected data on 15 phenotypic traits relevant to local adaptation in semi-arid regions. Data collection was carried out at appropriate growth stages, ensuring accurate and representative measurements for each trait. The heading date (HD, days) was recorded as the number of days from 1 January to the emergence of 50% of the spikes in a plot. Drought and heat tolerance were assessed based on the following physiological measurements: chlorophyll content (CC, CCI), canopy temperature (CT, °C), relative water content (RWC, %), and electrolyte leakage or leaf membrane thermostability (MT, %), which were determined at Zadoks growth stage (ZGS 55) [63] using appropriate laboratory techniques [64]. The morphological characteristics included flag leaf area (FLA, cm^2^), which was obtained at the heading date (ZGS 55), according to the procedure described by Spagnoletti-Zeuli and Qualset (1990) [65]. Plant height (PH, cm) was measured from the base of the plant to the tip of the spike at the maturity growth stage (ZGS 95). Yield components, such as spike number (SN, No m^−2^), spike weight (SW, t ha^−1^), grain yield (GY, t ha^−2^), biological yield (BY, t ha^−1^), and harvest index (HI, %), were recorded in each plot at the ZGS 95 growth stage after the harvest of the wheat samples from 2-row segments, 1 m long, across all of the replicates. Thousand kernel weight (TKW, g) was determined from the count and weight of 250 kernels. The number of grains per spike (NGS, No. spike^−1^) was derived from the grain number and spike number produced per square meter. 

### 4.6. Data Analysis

The collected phenotypic data were subjected to one-way analysis of variance (ANOVA) to assess the significance of differences among the cultivars for each recorded trait. Tukey’s honestly significant difference (HSD) at 5% and 1% levels of probability was employed to compare the means of the different cultivars. In parallel, the genetic and non-genetic parameters were calculated to explore the genetic variability within the plant material evaluated. This involved the computation of phenotypic (*CV_p_*), genotypic (*CV_g_*), and environmental (*CV_e_*) coefficients of variation, as described by Falconer and Mackay (1996) [66]. The *CV_p_* represents the total variation observed for a trait in the population, including both genetic and environmental factors. It was calculated as the standard deviation (*σ_p_*) divided by the mean (μ) of the trait, expressed as a percentage. The *CV_g_* estimates the proportion of total variation contributed by genetic factors alone, excluding environmental effects. It was calculated as the standard deviation of genotypic values (*σ_g_*) divided by the mean value (µ) of the trait, expressed as a percentage. The *CV_e_* represents the proportion of total variation attributed to environmental factors, excluding genetic effects. It was calculated as the standard deviation of the environmental values (*σ_e_*) divided by the mean (μ) of the trait, expressed as a percentage. Additionally, the *CV_g_/CV_e_* ratio was computed to assess the relative importance of genetic and environmental factors contributing to the observed phenotypic variation for measured traits within the durum wheat germplasm collection [67]. A higher *CV_g_*/*CV_e_* ratio indicates a greater contribution of genetic factors to the observed variation, suggesting that the trait is predominantly influenced by the genetic differences among individuals rather than the environmental conditions. Thus, this ratio provides breeders with valuable information to optimize breeding programs. It guides decisions on trait selection, breeding methodologies, and the development of varieties that perform consistently across different conditions. 

Broad-sense heritability (*h^2^_bs_*) provides an indication of the degree to which the observed variation in a trait is influenced by genetic factors, relative to environmental influences [66]. This statistical measure was obtained as the genetic variance (*σ^2^_g_*), divided by the phenotypic variance (*σ^2^_e_*) of the considered trait, expressed as a percentage. It is worth noting that these parameters are typically used in quantitative genetics and plant breeding research to understand the potential for genetic improvement and to make informed decisions in breeding programs [19,66]. 

Phenotypic (*r_p_*) and genotypic (*r_g_*) correlation coefficients were calculated based on the replicated data values to explore the relationships between different traits and to assess the potential for trait improvement through selection. Besides this, the *r_g_* values were divided into the direct and indirect genotypic effects of the 13 measured traits (independent variables) on grain yield (dependent variable), using the path analysis method, as outlined by Dewey and Lu (1959) [26]. The durum wheat cultivars were classified into clusters using Tocher’s optimization method [68]. The Mahalanobis (1936) distance was used to provide insights into the genetic structure and diversity within the durum wheat cultivars considering all recorded traits. To perform all of the mentioned statistical methods, ‘variability’ [69], ‘ggplot2’ [70], and ‘corrplot’ [71] statistical packages in R (Version 4.3.2) and Genes [67] software (Version 1990.2022.23) were used.

## 5. Conclusions

This study highlights the substantial genetic variation in durum wheat germplasm, offering promising prospects for effective breeding programs. Environmental influences contribute to observed trait variations alongside genetic effects. Traits such as canopy cover (CC), spike number (SN), spike weight (SW), thousand kernel weight (TKW), number of grains per spike (NGS), and harvest index (HI) exhibit significant positive correlations with grain yield at both genotypic and phenotypic levels, suggesting their importance in yield determination. The harvest index (HI) and spike number (SN) have the highest positive direct impact on grain yield, with substantial indirect effects through other yield components. Improvements in these traits could significantly increase yield per hectare. The identification of genetically diverse cultivars with desirable traits enables strategic crossbreeding to generate segregating progenies with maximum genetic variability, advancing durum wheat breeding programs. These findings contribute valuable insights into sustainable agriculture, particularly in semi-arid regions like Algeria, where environmental stresses pose significant challenges to durum wheat production.

## Figures and Tables

**Table 1:** Summary of the analysis of variance for the measured traits.

SV	Blocks	Genotypes	Residual	CV (%)
df	2	19	38	
HD	0.02	20.85 **	0.02	0.10
CC	19.19	203.57 **	50.52	16.32
CT	2.73	10.73 **	1.16	3.28
RWC	16.07	59.65 **	12.78	5.06
MT	70.30	340.29 ^ns^	183.79	16.81
FLA	10.51	38.54 **	11.09	11.00
PH	43.80	344.75 **	15.61	4.97
SN	5255.40	20,530.40 **	5001.5	18.68
SW	0.35	2.30 **	0.56	18.39
TKW	4.98	96.24 **	7.35	9.02
NGS	0.07	19.76 **	2.29	17.15
GY	0.03	1.23 **	0.23	24.91
BY	2.58	6.40 ^ns^	3.73	17.70
HI	1.87	113.56 **	11.28	18.76

SV: Source of variation, df: degrees of freedom, HD: Heading date (days), CC: Chlorophyll content (CCI), CT: Canopy temperature (°C), RWC: Relative water content (%), MT: Membrane thermostability (%), FLA: Flag leaf area (cm^2^), PH: Plant height (cm), SN: Spike number (No m^−2^), SW: Spike weight (t ha^−1^), TKW: Thousand kernel weight (g), NGS: Number of grains per spike (No spike^−1^), GY: Grain yield (t ha^−1^), BY: Biological yield (t ha^−1^), HI: Harvest index (%). ns and **: non-significant and significant at 1% probability level by *F*-test.

**Table 2:** Descriptive statistics for the measured traits in the wheat varieties.

Traits	Mean ± SE	Minimum	Maximum	HSD (5%)	HSD (1%)
HD	132.4 ± 0.07	128.0	138.0	0.40	0.47
CC	43.6 ± 4.10	29.3	54.3	22.07	25.61
CT	32.8 ± 0.62	29.7	36.9	3.34	3.88
RWC	70.6 ± 2.06	62.3	81.7	11.10	12.88
MT	80.6 ± 7.82	51.4	95.2	42.10	48.85
FLA	30.3 ± 1.92	21.9	36.3	10.34	12.00
PH	79.6 ± 2.28	69.3	108.7	12.27	14.24
SN	378.7 ± 40.83	225.0	496.7	219.62	254.85
SW	4.1 ± 0.43	2.3	5.2	23.15	26.87
TKW	30.1 ± 1.56	21.1	41.0	8.42	9.77
NGS	8.8 ± 0.87	4.3	15.8	4.70	5.45
GY	1.9 ± 0.28	1.1	3.2	1.49	1.73
BY	10.9 ± 1.11	7.2	12.3	6.00	6.96
HI	17.9 ± 1.93	9.6	32.1	10.43	12.10

HD: Heading date (days), CC: Chlorophyll content (CCI), CT: Canopy temperature (°C), RWC: Relative water content (%), MT: Membrane thermostability (%), FLA: Flag leaf area (cm^2^), PH: Plant height (cm), SN: Spike number (No m^−2^), SW: Spike weight (t ha^−1^), TKW: Thousand kernel weight (g), NGS: Number of grains per spike (No spike^−1^), GY: Grain yield (t ha^−1^), BY: Biological yield (t ha^−1^), HI: Harvest index (%).

**Table 3:** Estimates of genetic and non-genetic parameters for the measured traits in the wheat varieties.

Traits	*σ^2^_p_*	*σ^2^_g_*	*σ^2^_e_*	*CV_p_ (%)*	*CV_g_ (%)*	*CV_g_/CV_e_*	*h^2^_bs_*
HD	6.96	6.94	0.02	1.99	1.99	20.41	1.00
CC	101.54	51.02	50.52	23.14	16.40	1.00	0.50
CT	4.35	3.19	1.16	6.36	5.44	1.66	0.73
RWC	28.40	15.62	12.78	7.54	5.60	1.11	0.55
MT	235.96	52.17	183.79	19.05	8.96	0.53	0.22
FLA	20.24	9.15	11.09	14.86	9.99	0.91	0.45
PH	125.32	109.71	15.61	14.07	13.17	2.65	0.88
SN	10,177.76	5176.29	5001.47	26.64	19.00	1.02	0.51
SW	1.14	0.58	0.56	26.34	18.86	1.03	0.51
TKW	36.98	29.63	7.35	20.22	18.10	2.01	0.80
NGS	8.12	5.83	2.29	32.29	27.36	1.59	0.72
GY	0.57	0.33	0.23	38.95	29.92	1.20	0.59
BY	4.62	0.89	3.73	19.71	8.64	0.49	0.19
HI	45.37	34.09	11.28	37.63	32.62	1.74	0.75

HD: Heading date, CC: Chlorophyll content, CT: Canopy temperature, RWC: Relative water content, MT: Membrane thermostability, FLA: Flag leaf area, PH: Plant height, SN: Spike number, SW: Spike weight, TKW: Thousand kernel weight, NGS: Number of grains per spike, GY: Grain yield, BY: Biological yield, HI: Harvest index. *σ^2^_p_*: Phenotypic variance, *σ^2^_g_*: Genetic variance, *σ^2^_e_*: Environmental variance, *CV_p_*: Phenotypic coefficient of variation, *CV_g_*: Genotypic coefficient of variation, *CV_e_*: Environmental coefficient of variation, *h^2^_bs_*: Broad-sense heritability.

**Table 4:** Direct (diagonal) and indirect effects (off-diagonal) of 14 measured traits on grain yield in the wheat cultivars at a genotypic level.

Traits	HD	CC	CT	RWC	MT	FLA	PH	SN	SW	TKW	NGS	BY	HI
HD	**−0.047**	0.065	−0.145	0.167	0.078	−0.009	0.123	−0.239	0.111	0.004	−0.022	0.009	−0.200
CC	−0.010	**0.302**	−0.193	−0.054	0.060	−0.020	−0.002	0.104	−0.079	0.018	0.008	0.060	0.149
CT	0.018	−0.154	**0.380**	−0.133	−0.136	−0.002	−0.051	−0.042	0.051	−0.027	−0.045	−0.044	0.092
RWC	−0.021	−0.043	−0.133	**0.381**	0.007	−0.032	0.052	−0.049	0.038	−0.012	0.034	0.055	−0.644
MT	−0.022	0.107	−0.306	0.016	**0.169**	0.043	−0.008	−0.206	0.001	0.039	−0.073	−0.064	0.734
FLA	−0.005	0.072	0.008	0.146	−0.086	**−0.084**	−0.005	0.338	−0.063	−0.018	0.029	0.102	−0.221
PH	−0.027	−0.002	−0.090	0.092	−0.006	0.002	**0.215**	−0.209	0.087	0.022	0.037	0.025	−0.363
SN	0.022	0.062	−0.032	−0.037	−0.069	−0.056	−0.089	**0.503**	−0.159	−0.015	0.053	0.053	0.036
SW	0.025	0.114	−0.093	−0.069	−0.001	−0.025	−0.089	0.383	**−0.209**	0.013	0.075	0.029	0.116
TKW	−0.004	0.097	−0.186	−0.079	0.118	0.027	0.084	−0.135	−0.050	**0.055**	0.009	−0.019	0.415
NGS	−0.006	−0.013	0.094	−0.070	0.067	0.013	−0.043	−0.145	0.085	−0.003	**−0.183**	−0.029	0.925
BY	−0.005	0.206	−0.188	0.238	−0.122	−0.096	0.061	0.301	−0.070	−0.012	0.060	**0.088**	−0.413
HI	0.008	0.039	0.030	−0.210	0.106	0.016	−0.067	0.016	−0.021	0.020	−0.145	−0.031	**1.166**
*r_g_*	−0.105 ^ns^	0.343 **	−0.092 ^ns^	−0.365 **	0.431 **	0.212 ^ns^	−0.220 ^ns^	0.272 *	0.270 *	0.331 **	0.693 **	0.048 ^ns^	0.925 **

HD: Heading date, CC: Chlorophyll content, CT: Canopy temperature, RWC: Relative water content, MT: Membrane thermostability, FLA: Flag leaf area, PH: Plant height, SN: Spike number, SW: Spike weight, TKW: Thousand kernel weight, NGS: Number of grains per spike, GY: Grain yield, BY: Biological yield, HI: Harvest index, *r_g_*: Genotypic correlation coefficient with grain yield. ns, * and **: non-significant and significant at 5% and 1% probability levels by *t*-test.

**Table 5:** Clustering pattern of the durum wheat cultivars based on measured traits using Tocher’s method.

Cluster No.	No. of Cultivars	Contribution (%)	Cultivars
G1	8	40	Waha, Simeto, Amar 06, Boutaleb, Saoura, Wahbi, Manssourah, Massinissa
G2	4	20	Megress, Mexicali75, Zb/Fl, Ain Lehma
G3	3	15	Oued El Bared, Belikh02, Ofanto
G4	2	10	Mohamed Ben Bachir, Guemgoum Rkhem
G5	1	5	Setifis
G6	1	5	Bousselam
G7	1	5	Vitron

**Table 6:** Cluster mean values for 14 studied traits in the durum wheat cultivars.

Traits	Clusters						
	G1	G2	G3	G4	G5	G6	G7
HD	133.5	130.7	128.7	137.5	135.0	132.0	130.0
CT	47.2	46.9	39.1	45.2	30.0	29.3	38.9
MT	32.2	32.8	34.4	31.9	32.3	36.9	31.5
CC	71.0	71.0	67.0	75.4	69.8	71.7	67.5
RWC	84.7	81.3	69.7	79.3	91.2	64.8	86.3
FLA	32.2	30.6	28.1	28.8	25.1	33.8	24.7
PH	78.5	74.1	76.4	104.7	73.3	76.7	78.0
SN	408.8	411.3	373.9	240.8	288.3	408.3	358.3
SW	4.4	4.3	3.9	2.4	3.2	3.8	5.0
TKW	31.9	28.9	27.7	30.6	25.2	21.1	40.1
NGS	9.6	7.6	10.0	8.4	10.1	7.5	4.3
GY	2.4	1.8	1.9	1.1	1.5	1.3	1.2
BY	11.6	11.0	10.5	11.1	8.1	11.0	9.2
HI	21.2	16.6	19.7	9.8	18.2	11.6	13.6

HD: Heading date (days), CC: Chlorophyll content (CCI), CT: Canopy temperature (°C), RWC: Relative water content (%), MT: Membrane thermostability (%), FLA: Flag leaf area (cm^2^), PH: Plant height (cm), SN: Spike number (No m^−2^), SW: Spike weight (t ha^−1^), TKW: Thousand kernel weight (g), NGS: Number of grains per spike (No spike^−1^), GY: Grain yield (t ha^−1^), BY: Biological yield (t ha^−1^), HI: Harvest index (%).

**Table 7:** Plant material evaluated in the field experiment.

N°	Name	Origin	Pedigree
1	Mohamed Ben Bachir	Algeria	Local landrace
2	Bousselam	CIMMYT-ICARDA	Heider/Martes/Huevos de Oro. ICD-414
3	Oued El Bared	Algeria	Gta Dur/Ofanto
4	Waha	CIMMYT-ICARDA	Plc/Ruff//Gta/Rtte
5	Boutaleb	Algeria	Hedba03/Ofanto
6	Saoura	ACSAD	Belikh//Gediz/Bit
7	Megress	Algeria	Ofanto//Waha/Mohamed Ben Bachir
8	Manssourah	Algeria	Chinese spring/Mohamed Ben Bachir
9	Vitron	Spain	Turkey77/3/Jori/Anhinga//Flamingo
10	Amar06	ICARDA	Lgt3/Bicre// cham1//orlgt3/4/Bicre/3/Ch1//Gav/Starke
11	Wahbi	Algeria	Bidi17/Waha//Bidi17
12	Simeto	Italy	Capeiti8/Valnova
13	Ain Lehma	ICARDA	Bcr/Gro1//Mgn/1
14	Massinissa	Algeria	Ofanto/Bousselam
15	Setifis	Algeria	Bousselam/Ofanto
16	Belikh02	ICARDA	Crane/Stork
17	Mexicali75	CIMMYT	Gdovz469/3/Jo’s’//61.130/Lds
18	Zb/Fl	CIMMYT	Zb/Fg’s//Lk/3/Ko120/4/Ward
19	Ofanto	Italy	Appulo/Adamello
20	Guemgoum Rkhem	Algeria	Local landrace

ICARDA: International Center for Agricultural Research in the Dry Areas, CIMMYT: International Maize and Wheat Improvement Center, ACSAD: Arab Center for the Studies of Arid Zones and Dry Lands.

**Table 8:** Soil characteristics at the Setif ITGC-FDPS experimental site.

Parameters	Values
Total CaCO_3_ (%)	35.01
pH	8.21
CEC (meq 100 g^−1^)	25.0
EC (mmhos cm^−1^)	0.21
OM (%)	1.97
Total N (‰)	1.01
P_2_O_5_ (ppm)	103.82
K_2_O (ppm)	588.7

## Data Availability

Data are contained within the article.
